# The transcription factor CpMYB62 controls the genetic network that leads to the determination of female flowers in *Cucurbita pepo*

**DOI:** 10.1093/hr/uhae115

**Published:** 2024-04-22

**Authors:** María Segura, Alicia García, German Gamarra, Álvaro Benítez, Jessica Iglesias-Moya, Cecilia Martínez, Manuel Jamilena

**Affiliations:** Department of Biology and Geology. Agri-food Campus of International Excellence (CeiA3) and Research Center CIAIMBITAL, University of Almería, 04120 Almería, Spain; Department of Biology and Geology. Agri-food Campus of International Excellence (CeiA3) and Research Center CIAIMBITAL, University of Almería, 04120 Almería, Spain; Department of Biology and Geology. Agri-food Campus of International Excellence (CeiA3) and Research Center CIAIMBITAL, University of Almería, 04120 Almería, Spain; Department of Biology and Geology. Agri-food Campus of International Excellence (CeiA3) and Research Center CIAIMBITAL, University of Almería, 04120 Almería, Spain; Department of Biology and Geology. Agri-food Campus of International Excellence (CeiA3) and Research Center CIAIMBITAL, University of Almería, 04120 Almería, Spain; Department of Biology and Geology. Agri-food Campus of International Excellence (CeiA3) and Research Center CIAIMBITAL, University of Almería, 04120 Almería, Spain; Department of Biology and Geology. Agri-food Campus of International Excellence (CeiA3) and Research Center CIAIMBITAL, University of Almería, 04120 Almería, Spain

## Abstract

In monoecious species, female flowering constitutes the developmental process that determines the onset and production of fruit and is therefore closely related to crop yield. This article presents the identification and phenotypic and molecular characterization of *myb62*, an ethylmethane sulfonate loss-of-function mutation that completely blocks the female floral transition, converting all female flowers into male flowers. BSA-seq analysis coupled with WGS showed that *myb62* corresponds to a C>T transition in the coding region of the gene *CpMYB62*, generating a premature stop codon and a truncated transcription factor without its N-terminal effector domain. The *myb62* phenotype was partially rescued by exogenous ethylene application, indicating that the function of *CpMYB62* is mediated by ethylene. Different evidence supports this conclusion: first, the reduced ethylene production of the mutant, and second, the male flower productive phenotype of the double mutant between *myb62* and the ethylene-insensitive mutant *etr2b*, which demonstrated that *myb62* is epistatic over *etr2b*. Furthermore, transcriptomic analysis of WT and *myb62* apical shoots confirmed that *CpMYB62* regulates master sex-determining genes, upregulating those encoding the ethylene biosynthesis enzymes *CpACO2B* and *CpACS27A* and those encoding for transcription factors that promote the development of carpels(*CpCRC*), but downregulating those involved in the arrest of carpels (*CpWIP1*), In the gene network controlling sex determination in cucurbits, CpMYB62 occupies the most upstream position, activating ethylene and other sex determining genes involved in female flower determination in *Cucurbita*  *pepo*.

## Introduction

The Cucurbitaceae family comprises several economically significant horticultural crops, including cucumber (*Cucumis sativus*), melon (*Cucumis melo*), watermelon (*Citrullus lanatus*), pumpkin (*Cucurbita maxima*) and squash (*Cucurbita pepo*) [1]. The cultivated species of Cucurbitaceae are monoecious (unisexual male and female flowers in the same individual plant) or dioecious (unisexual male and female flowers in separated plants), but some relative species are also hermaphrodite (only hermaphrodite flowers). Squashes and pumpkins are classified in the genus *Cucurbita*, whose plants are predominantly monoecious, meaning they develop male and female flowers on the same individual plant [2]. The distribution of unisexual male and female flowers in the plant distinguishes three sexual phases of development: (i) the early androecious phase, where only male flowers are produced; (ii) the mix phase of development, which occurs after female flower transition and where the plant alternates the production of male and female flowers, and (iii) a late gynoecious phase, which occurs only in some cultivars under certain environmental conditions and where the plant produces only female flowers [3].

Sex determination mechanisms in cucurbits are dependent on environmental, hormonal, and genetic factors [3], all converging on the central player, the gaseous hormone ethylene [2, 4]. External application of ethylene has a feminizing effect on *C. sativus, C. melo, C. lanatus, C. maxima*, and *C.pepo* [5–8], accelerating female flowering transition and increasing the number of female flowers per plant. Similarly, external treatments with brassinosteroids [8] and auxins [9], also have a feminizing effect, which seems to be mediated by ethylene. Conversely, the application of gibberellin (GA) has a masculinizing effect on cucurbits plants, although whether this effect is mediated by ethylene is not entirely clear yet [10, 11].

The role of ethylene in sex determination is widely reported in cucurbits. Mutations in different ethylene related genes were found to promote changes in floral sex phenotypes and in the duration of sexual phases of development. The ethylene biosynthetic genes *ACS11* and *ACO2* are necessary for carpel development in *C. melo* and *C. sativus* [12, 13]. Mutants for *ACS11* and *ACO2* are androecious (plants that produce only male flowers). Female flower determination also requires the expression of the ethylene biosynthesis gene *CpACS27A* of squash, *CsACS2* of cucumber, *CmACS7* of melon, and *CitACS4* of watermelon, as well as the gene *CpACO1A* of squash, which are necessary to suppress the development of stamens in female flowers [14–18]. Mutants for these orthologous genes are andromonoecious, producing male and hermaphrodite flowers on the same individual plant. Ethylene receptors are also required for both carpel promotion and stamen arrest during the development of female flowers, and mutants for three different receptors in *C. pepo* result in androecy (plants that produce only male flowers) or andromonoecy (plants that produce male and hermaphrodite flowers) [19, 20]. Ethylene receptors not only activate the signaling cascade initiated by *ACS11* and *ACO2*, and *ACS27A* and *ACO1A* in the female flowers [21] but also the feedback regulation of these ethylene biosynthesis genes in the floral meristem [22, 23].

The molecular mechanisms that regulate sex determination upstream and downstream of ethylene are not so well known. In melon, cucumber, and watermelon, the transcription factor WIP1, which is repressed by ethylene biosynthesis genes *ACS11* and *ACO2*, is crucial for preventing carpel development during the specification of the male flower [24–26]. On the other hand, WIP1 performs its action by repressing both the homeotic gene *CRABS CLAW* (*CmCRC*) which is involved in carpel development in both Arabidopsis [27] and melon [28], as well as the melon ethylene biosynthesis gene *CmACS7*, suppressing so the arrest of stamen that led to the male flower specification in melon [28]. In the female flower, however, the suppression of *WIP1* and the induction of *CmACS7* lead to the expression of the transcription factor gene *HB40,* and finally to the arrests of stamen development [29]. In *C. pepo*, the transcription factor *CpCUC2B* has been recently reported as a repressor of carpel development in an ethylene-independent manner. *CpCUC2B* also promotes stamen development by inhibiting *CpACS27A* [30].

Despite the progress in weaving the genetic network that regulates sex determination of cucurbits, no factor has yet been found that regulates ethylene production and signaling for the specification of female flowers. In this study, we identify and characterize a mutation in the transcription factor gene *CpMYB62* that results in the conversion of female into male flowers, and monoecy into androecy in *C. pepo*. CpMYB62 acts upstream of ethylene production, shedding light on the early mechanisms promoting ethylene production and carpel development during the determination of the female flower.

## Results

### *myb62* converts monoecy into androecy

**Figure 1:**
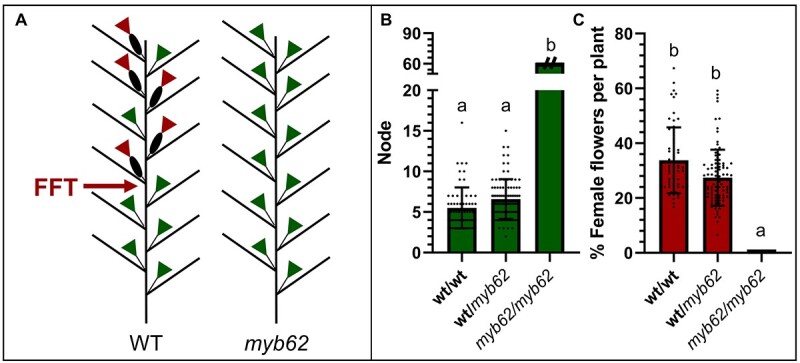
Sex phenotype of *myb62*. (**A**) Schematic representation of the distribution of male and female flowers in WT and *myb62* mutant plants. Green = male flower; brown = female flower. (**B**, **C**) Production of female flowers in WT and heterozygous and homozygous plants for the *myb62* mutation. (**B**) Node of the occurrence of the first female flower (green bars). (**C**) Percentage of female flowers per plant (brown bars) in plants with a minimum of 60 nodes. The error bars represent SE. Different letters indicate significant differences between plant genotypes (ANOVA, *P* ≤ 0.05). The dots indicate individual values. None of the mutant plants *myb62/myb62* showed female flower production in the first 60 nodes of the plant.

The *myb62* mutant line was identified in a screening of 123 mutant lines from the ethylmethane sulfonate (EMS) collection of *C. pepo* in the genetic background MU-CU-16 [31]. More than 8% of the mutant lines showed alteration in flower development (Supplementary Table S1), and one of them, named *myb62*, conferred an androecious phenotype, with a complete absence of female flower production after evaluating more than 60 nodes (Fig. 1A, Supplementary Table S2). M2 mutant plants were crossed with the scallop line UPV-196 to obtain the F1 and F2 generations and backcrossed for twice with the parental line MU-CU-16 to obtain the BC1 and BC2 populations. The latter populations were then selfed to obtain the BC1S1 and BC2S1 segregating populations. Sex phenotyping was performed during the spring–summer seasons of 2021 and 2022 on 99 F2 plants and 209 BC2S1 plants (Supplementary Table S2). This evaluation allowed us to confirm the effect of the *myb62* mutation in two different genetic backgrounds, UPV-196 and MU-CU-16. The generations F1, BC1, and BC2 showed the monoecious phenotype of WT plants, and the F2 and BC2S1 populations segregated 3:1 for monoecious:androecious. These results indicate that *myb62* is a recessive mutation (Supplementary Table S2, Fig. 1B). The male flowers of the androecious *myb62* plants developed normally and were completely functional (Supplementary Fig. S1). Therefore, the effect of *myb62* was restricted to the conversion of females into male flowers.

**Table 1:** Phenotypic evaluation of single and double mutants between *myb62* and the ethylene-insensitive mutant *etr2b*

Genotype	Female flowering transition	% pistillate flowers	% bisexual flowers*	Phenotype
**WT**	11.88 ± 1.66 a	21.55% ± 1.89% c	0.00% ± 0.00% a	Monoecious
***myb62***	-	0.00% ± 0.00% a	-	Androecious
***etr2b***	14.14 ± 1.85 a	8.63% ± 1.58% b	6.21% ± 1.14% b	Partially andromonoecious
***myb62*/*etr2b***	-	0.00% ± 0.00% a	-	Androecious

^*^Pistillate flowers with developed stamens from the total of flowers analyzed of each genotype.

Significant differences are indicated by different letters in the same column (ANOVA, p ≤ 0.05).

### *myb62* mutation affects the effector domain of the *CpMYB62* transcription factor gene

**Figure 2:**
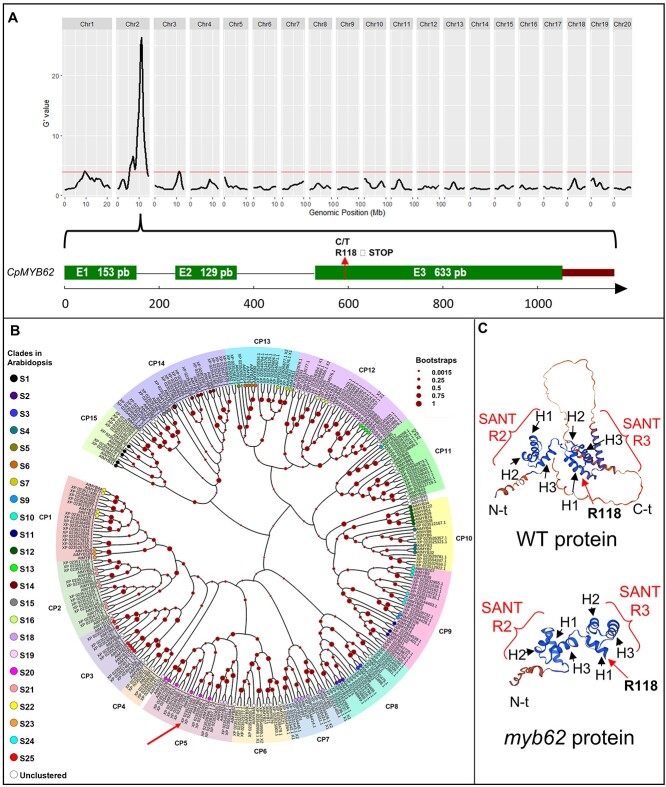
Identification of *myb62* causal mutation by BSA-seq coupled with QTL-seq. (**A**) QTL analysis of WT and *myb62* bulks by QTLseqr. G’-value is represented in Y-axis. Chromosome interval in represented in X-axis. The red line represents the threshold line with a confidence level of 0.99. The QTL analysis shows a region in chromosome 2 linked with the mutant phenotype. After filtering, a punctual mutation in the exon 3 of a MYB gene (*Cp4.1LG02g13220*) causing a premature STOP codon was selected as responsible of the phenotype. (**B**) Phylogenetic relationship of MYB transcription factor identified in *Cucurbita pepo* and *Arabidopsis thaliana*. There are 15 branches in *C. pepo*, corresponding to clades CP1 to CP15. Branches with dots indicates Arabidopsis genes. Different colors indicate the different clades in Arabidopsis according to [32]. CpMYB62 protein is indicated with a red arrow in clade CP5, clustering together with AtMYB62. (**C**) Structural prediction of WT and mutant CpMYB62 protein. The mutant protein is missing the whole C-terminal (C-t) effector domain.

For the identification of the causal mutation of the *myb62* androecious phenotype, WT and mutant plants in the F2 offspring of *myb62* x UPV-196 were subjected to Bulk Segregant Analysis Sequencing (BSA-seq). The obtained reads covered 98% of the reference genome with an average depth of 39.51 reads in WT-bulk, and 48.31 reads in *myb62*-bulk (Supplementary Table S3). More than 1.5 million single nucleotide polymorphisms (SNPs) were found in the two bulks. The quantitative trait locus (QTL)-Seq analysis allowed the identification of two adjacent significant QTLs in chromosome 2 (Supplementary Table S4). The Δ(SNP-index) values in that region were close to 0.7, indicating that this region contains the *myb62* locus (Fig. 2A, Supplementary Fig. S2).

Variants within QTLs in chromosome 2 were filtered out, selecting EMS canonical changes (C > T or G > A) and those variants with an alternative AF of 1 in the mutant bulk, and maximum AF of 0.30 in the WT bulk. From the 101 selected SNPs, only 20 were exonic and 3 of them were specific to the line containing the *myb62* mutation (SNP1–3; Supplementary Table S3). SNP1, SNP2, and SNP3 disrupt a *MYB transcription factor*; a *WPP domain-interacting tail-anchored*, and a *phosphoinositide phospholipase C*, respectively (Supplementary Table S3). A fine mapping analysis was finally performed to select the causal mutation of the *myb62* phenotype. The three putative SNPs were genotyped in 248 plants from *myb62* segregating populations (F2, BC1S1 and BC2S1). Only the mutation SNP1 in the MYB transcription factor co-segregated 100% with the *myb62* phenotype. The SNP2 and SNP3 segregated from the phenotype in 8% and 2% of the plants (Supplementary Table S5). The genotyping of 231 additional plants confirmed the perfect co-segregation in a total of 479 plants. We assumed therefore that the androecious *myb62* phenotype was caused by the EMS mutation found in the *MYB transcription factor* gene. This was a C > T transition at the position 598 of the genomic sequence, which generated a premature STOP codon in the residue 118 of the protein (Fig. 2A).

The phylogenetic analysis between *C. pepo* and Arabidopsis MYB proteins indicated that the identified MYB factor in chromosome 2 (*Cp4*.*1LG02g13220*; NCBI: XP_023523160), cluster together with another *C. pepo* MYB in chromosome 13 (*Cp4.1LG13g04850*, NCBI: XP_023551169) and the Arabidopsis AtMYB62 (Fig. 2B). So, the squash MYB on chromosome 2 was named CpMYB62, and the other CpMYB62-A. The two squash MYB proteins share a 55% identity, but they do not appear to be paralogs, since they were found to be located in different synteny blocks (Supplementary Table S6). CpMYB62 is an R2R3 type MYB factor, the largest subgroup within this type of transcription factors. This group is characterized by having two consecutive repeats of the SANT-type domain at the N-terminal, responsible for DNA recognition and binding, and an effector or transactivator domain at the C-terminal that modulates transcription factor activity [32]. *myb62* is a STOP gain mutation that falls into amino acid 118 of the second SANT domain, resulting in a truncated protein that lacks the complete C-terminal effector domain (Fig. 2C).

### Hormonal treatments rescuing the *myb62* mutant phenotype

Given that ethylene and GAs regulate sex determination in squash, promoting the development of female or male flowers, respectively, we tried to rescue the *myb62* phenotype by treating mutant plants with either paclobutrazol or ethylene. Moreover, it is reported that Arabidopsis *AtMYB62* regulates GA biosynthesis and signaling pathway [33]. WT and *myb62* plants were subjected to the application of exogenous gibberellic acid (GA3) and paclobutrazol, determining the effect of these treatments on plant growth and sex expression. The data showed that the application of exogenous GA3 and paclobutrazol resulted in either an increase or a reduction in plant internode length in both WT and *myb62* (Supplementary Fig. S3). Similarly, GA3 and paclobutrazol led to a significant decrease or increase in the ratio of female flowers per plant in the WT (Supplementary Table S7). However, the production of female flowers in *myb62* plants was unaffected by any of the treatment, indicating that the androecious phenotype of *myb62* was not caused by an increase in GA.

**Figure 3:**
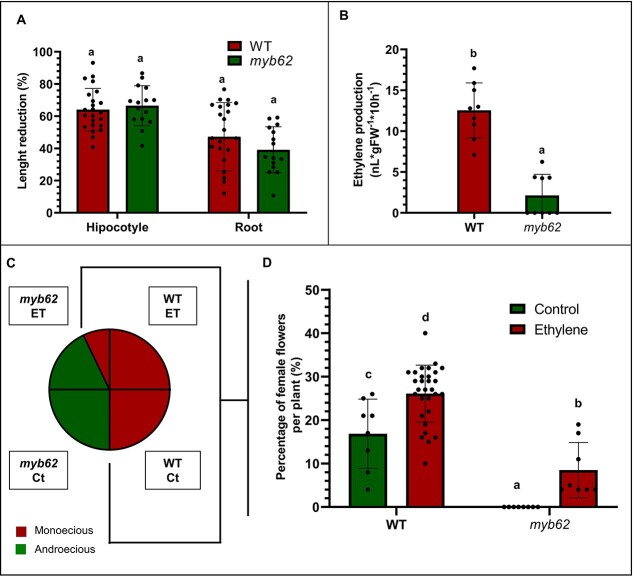
Ethylene rescues the mutant phenotype of *myb62*. (**A**) Ethylene sensitivity of *myb62* assessed by the ethylene triple response of etiolated seedlings. The percentage of reduction in hypocotyl and root length produced by ethylene in WT and *myb62* seedlings is shown. (**B**) Ethylene production in WT and *myb62* apical shoots at M1 stage. (**C**) Effect of external ethylene application on the percentage of monoecious and androecious plants in WT and *myb62* plants. Ct: Control plants; ET: Ethylene-treated plants. (**D**) Effect of ethylene on female flowers production of WT and *myb62* plants. The error bars indicate SE. Different letters showed significant differences between plant genotypes and treatment (ANOVA, *P* ≤ 0.05). The dots indicate individual values.

Regarding ethylene, we analyzed the effect of *myb62* on ethylene sensitivity using the triple response assay. The ethylene-induced reduction in the length of hypocotyl and the root in darkness was similar in the WT and *myb62* plants, indicating that both genotypes had the same ethylene sensitivity (Fig. 3A). In contrast, ethylene production was remarkably reduced in *myb62* apical shoots from adult plants (Fig. 3B). We also assessed the effect of external application of ethylene on WT and *myb62* plants sex expression. A gaseous ethylene treatment induced female flower production not only in the monoecious WT but also in the androecious *myb62* plants. Ethylene increased the ratio of female flowers per plant from 17% to 26% in the WT (Fig. 3D). On the other hand, ethylene converted 29% of the androecious *myb62* plants into monoecious (Fig. 3C), with an average percentage of female flowers per plant of 8% (Fig. 3D). Interestingly, ethylene changed from male to female flowers in the nodes after the treatment, but later the plant reverted to the production of male flowers (Supplementary Fig. S4). This partially rescue phenotype suggests that the androecy of the mutant is mediated by a deficiency in ethylene and it is the external application perceived by *myb62* plants that induced the production of female flowers. In fact, the ethylene triple response test indicated that *myb62* plants perceived and responded to ethylene as WT. These also mean that *CpMYB62* controls sex determination by acting upstream of the ethylene biosynthesis network, rather than in the perception or signaling pathway. Consequently, we studied the interaction between ethylene and *myb62* by generating and phenotyping the double mutant *myb62/etr2b*. The ethylene insensitive *etr2b* is a gain-of-function mutation that showed partial andromonoecious phenotype [20], in which 6.21% of the female flowers developed stamen and were converted into bisexual flowers (Table 1). However, the double mutant *myb62/etr2b* showed the androecious phenotype of *myb62* (Table 1), demonstrating that both genes are required for the development of female flowers and that *myb62* is epistatic over *etr2b*. Given that *myb62* reduced ethylene production, and that the external application of ethylene rescued the mutant phenotype, we concluded that the action of *CpMYB62* in the regulation of female flower specification occurs upstream of *CpETR2B*.

**Figure 4:**
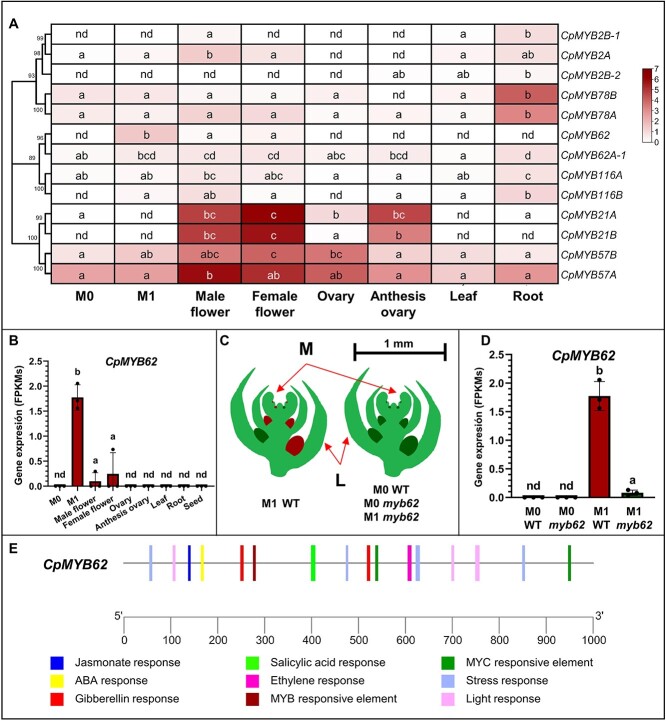
Expression patterns and regulation of the *CpMYB62* and *MYB* genes of the same clade. (**A**) Expression of *CpMYB* genes from clade CP5 and (**B**) expression of *CpMYB62* in different tissues of WT plants: M0 (male apical shoot from plants having 2–3 leaves; before female flowering transition), M1 (female apical shoot from plants with 12–14 leaves, after female flowering transition), male and female individual flowers with a corolla length of 30 mm, and ovaries from flowers at the same developmental stage, leaves of 10 mm, roots from plantlets of 17–21 days after germination and dry seeds. FPKM values were used to generate the heatmap and graph with hierarchical clustering analysis. **(C)** Schematic representation of 1 mm long apical shoots collected to perform RNA-seq analysis including the shoot apical meristem (M), leaf primordium (L) and floral buds with less than 0.5 mm (male, green; female, brown). The stages of plant development are indicated for WT and *myb62* mutant plants: stage M0, plants with 2–3 true leaves, and stage M1, plants with 12–14 true leaves. (**D**) Gene expression of *CpMYB62* in apical shoots of WT and *myb62* plants at M0 and M1 stages of development. FPKM values were used to generate the graph. The error bars indicate SE. Different letters indicate significant differences of each gene between analyzed tissues (ANOVA, *P* ≤ 0.05). nd, non-detected expression. The dots indicate individual values (**E**). Analysis of cis regulatory elements in the 1 Kb promoter region of *CpMYB62.* Elements related with hormones, stresses, and transcription factors are shown.

**Figure 5:**
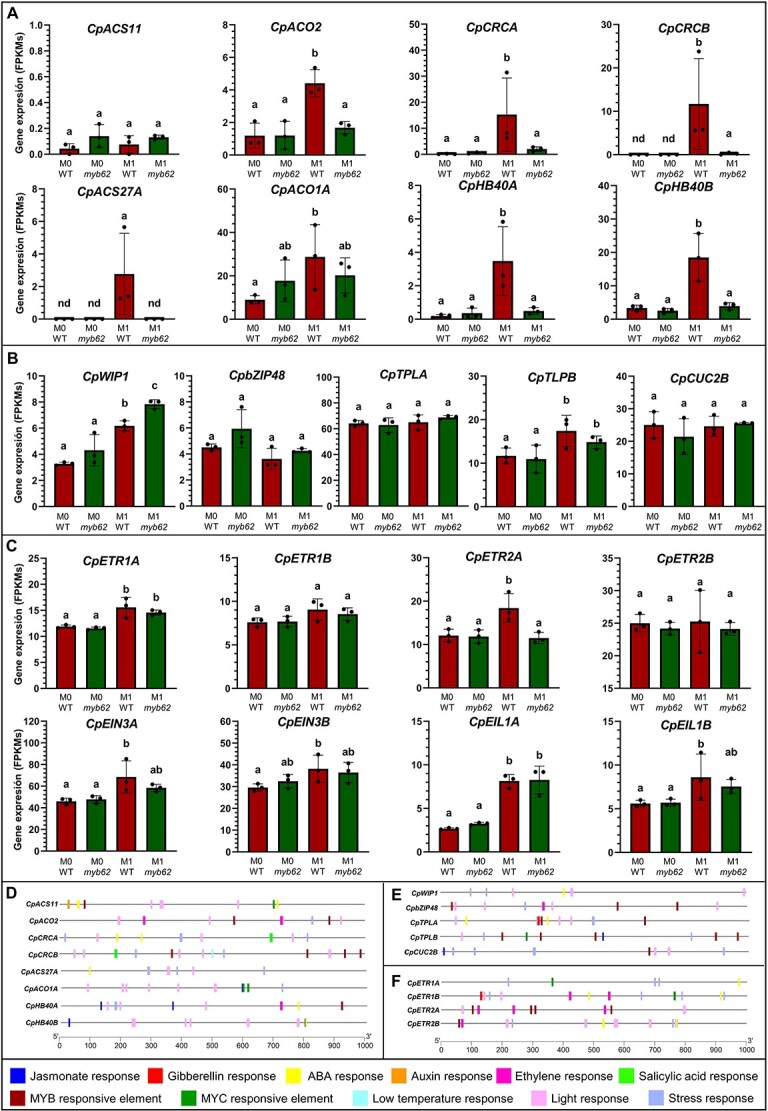
Expression pattern and regulation of known sex determination genes in the apical shoots of WT and *myb62* mutant plants at M0 (male producing) and M1 (male and female producing) stages of development. (**A**) Expression of ethylene biosynthesis and other genes involved in the promotion of carpel development, and the arrest of stamen development in female flowers. (**B**) Expression of genes involved in the arrest of carpel development in male flowers. (**C**) Expression of ethylene perception and signaling genes. FPKM values were used to generate the graphs and are represented on the Y-axis. The error bars represent SE. Significant differences of each gene between analyzed tissues are indicated by different letters (ANOVA, *P* ≤ 0.05). nd, no detectable expression. The dots indicate individual values. (**D–F**) Analysis of cis regulatory elements in the 1 Kb promoter region of sex determining genes. Elements related with hormones, stresses, and transcription factors are shown.

**Figure 6:**
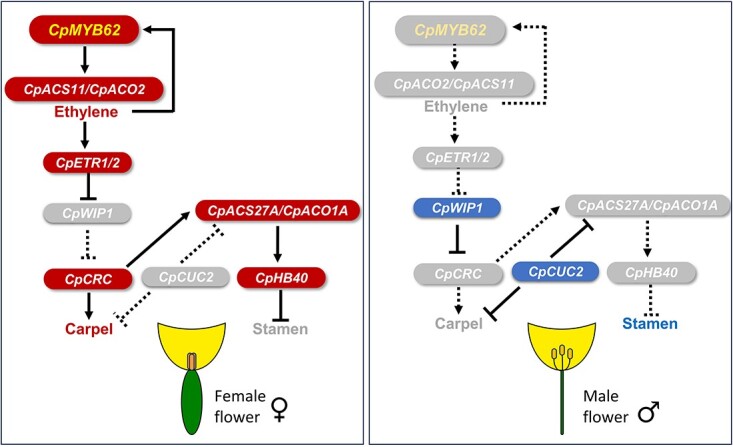
Gene network controlling the specification and development of female and male flowers in cucurbits. *CpMYB62* is the first reported sex determining gene that functions upstream of ethylene production. This transcription factor activates the ethylene biosynthesis genes and the downstream transcription factors that promote the development of carpels and arrest the development of stamen, resulting in the specification of a female flower. In the absence of CpMYB62 and ethylene, the male determining transcription factors CpWIP1 and CpCUC2 arrest the development of carpels, resulting in the specification of a male flower.

### Transcriptomic analysis of *CpMYB62*

To gain insight into the role of *CpMYB62* gene in female flowering promotion in *C.pepo*, we used the RNA sequencing (RNA-seq) project PRJNA1042934 to determine the expression of *CpMYB62* and 13 other related MYB genes of the same phylogenetic clade in different plant tissues of the background genotype MU-CU-16, including medium size and anthesis ovaries, roots, leaves, and dry seeds, but also the apical shoots of plant at the male (M0, before female flowering transition) and the female (M1, after female flowering transition) stages of development (Fig. 4A). The MYB genes showed a specific expression pattern in multiple plant tissues, but the only gene that was differentially expressed in male and female producing apical shoots was *CpMYB62*, stage identified as M1 (Fig. 4A). The gene *CpMYB62* had a very specific expression pattern, with no expression in vegetative organs and ovaries, and low expression in developed male and female flowers. The gene was not expressed in the apical shoot during the male phase of development but was induced in the apical shoot after the transition to female flowering (Fig. 4B).

An RNA sequencing study was performed to study the transcriptomic changes that occur in the apical shoot after female flowering transition (PRJNA1018819). The transcriptome of the apical shoots at the developmental stage M0 (plants with 2–3 leaves in the male phase of development) and M1 (plants with 12–14 leaves in the female flowering phase of development) was compared in WT and *myb62* plants (Supplementary Fig. S5). Since the transition in WT is observed between nodes 6 and 10, M0 corresponds to a developmental stage of the apical shoot with only male flower buds, while M1 corresponds to apical shoots with both female and male flower buds (Fig. 4C). The quality and mapping results for three replications per sample are shown in Supplementary Table S8. After cleaning, more than 20 M clean reads were obtained for all samples, with an average Q30 percentage of almost 92%. The average mapping rate against the reference genome MU-CU-16 (*C. pepo* Genome v4.1) was 94.5% (Supplementary Table S8). The principal component analysis (PCA) showed the consistency of the three biological replicates of each sample. PC1 and PC2, explained 55% and 9% of gene expression variation, with PC1 separating samples according to developmental stage (M0 vs. M1), and PC2 according to genotypes (WT vs. *myb62*) (Supplementary Fig. S6A). To validate the consistency of the results, we performed quantitative real-time polymerase chain reaction (qRT-PCR) for two ethylene biosynthesis genes, *CpACS11* and *CpACS27A*, which showed a similar expression profile by either RNA-seq or qRT-PCR (Supplementary Fig. S6B-C).

Given that the apical shoot comprises the apical meristem, together with small leaves and flower buds at the earliest stages of development, the WT apical shoots at stages M0 and M1 of development are differentiated only because those in M0 contain male floral buds, and those in M1 contain both male and female floral buds (Fig. 4C, Supplementary Fig. S5). RNA sequencing confirmed that the *CpMYB62* transcripts accumulated only in the apical shoots of WT plants in the M1 stage, those with female floral buds (Fig. 4D). In fact, the accumulation of *CpMYB62* in *myb62* plants in M1 was almost undetectable (Fig. 4D). These results, together with the *myb62* phenotype of *myb62* and the analysis of the impact of mutation on the ethylene pathway, show that *CpMYB62* plays an essential role in the developmental program that specifies the development of female flowers. Furthermore, we performed an analysis in the 1Kb promoter region of the *CpMYB62* gene to detect putative cis-regulatory elements, revealing several hormone-responsive sites, including ethylene, in the *CpMYB62* promoter (Fig. 4E).

### *CpMYB62* regulation of sex determining genes

The possible *CpMYB62* regulation of known sex determining genes, including those regulating the biosynthesis and signaling of ethylene, was studied by comparing the transcriptomes of WT and *myb62* apical shoots at M0 and M1 stages of development (Fig. 5). At the M0, when WT and *myb62* plants were both producing male flowers, none of the sex determining genes were differentially expressed. At the M1 stage of development, when WT plants were producing female and male flowers, but *myb62* plants were still at the male phase of development, a number of sex-determining genes were differentially expressed between WT and the mutant (Fig. 5). A number of the genes promoting the development of the carpel (*CpACO2*, *CpCRCA*, *CpCRCB*) and arresting the development of stamens (*CpACS27A*, *CpHB40A,* and *CpHB40B*) for the specification of the female flower were all downregulated in the *myb62* apical shoot at M1 (Fig. 5A). By the contrast, the gene CpWIP1, which participates in the arrest of carpel development for the specification of the male flower, was upregulated in the *myb62* apical shoot at M1 in comparison with WT (Fig. 5B). Among ethylene receptor genes, only the gene *CpETR2A* was downregulated by the *myb62* mutation at M1 (Fig. 5C). Other sex determining genes involved in the same processes, including the ethylene biosynthesis genes *CpACS11* and *CpACO1A*, the ethylene receptor genes *CpETR1A*, *CpETR1B,* and *CpETR2B*, ethylene signaling genes *CpEIN3A, CpEIN3B, CpEIL1A,* and *CpEIL1B*, and the transcription factor genes *CpCUC2B*, *CpbZIP48*, *CpTPLA*, *CpTPLB*, were not found to be regulated by the transcription factor *CpMYB62* (Fig. 5).

Finally, we searched for MYB responsive elements in 1 Kb promoter region of these sex determining genes. In addition to other cis-regulatory elements, MYB binding elements were found in genes involved in carpel development, including *CpACS11*, *CpACO2*, *CpCRCB*, *CpbZIP48*, *CpTLPs*, and *CpCUC2B*, in the stamen arrest gene *CpHB40A*, and in the ethylene receptors *CpETR2*A and *CpETR2B* (Fig. 5D-F ).

## Discussion

The study of an EMS mutant collection of the monoecious species *C. pepo* has facilitated the identification of novel components of the sex determination pathway in cucurbits [18–20, 30, 31, 34]. This article reports a mutation in the transcription factor *CpMYB62* that leads to the conversion of female flowers into male flowers, and monoecy into androecy. Therefore, the transcription factor is essential for the specification of female flowers in *C. pepo*. This is a novel function for MYB-type transcription factors, which are known to be involved in numerous plant developmental and physiological processes [32, 35]. CpMYB62 was found to be clustered in the CP5 clade in *C. pepo* (Fig. 2B), encompassing proteins from the Arabidopsis S19 and S20 clades [32]. Clade S19 includes AtMYB21, AtMYB24, and AtMYB57, which play a pivotal role in stamen development through the mediation of hormones such as GA and jasmonic acid (JA) [36–38]. Clade S20 includes AtMYB62, which regulates flowering genes through the action of GA [33]. Similarly to that found in Arabidopsis genes encoding MYB proteins in clade S20, transcriptomic data in *C. pepo* demonstrated that *CpMYB62* was specifically upregulated in female flowers at very early stages of development (Fig. 3B and C).

Ethylene is the key sex determining hormone in cucurbits, promoting the development of carpels and arresting the development of stamen during the specification of female flowers. GA also participates in cucurbit sex determination mechanisms by promoting the development of male flowers [3]. Since *AtMYB62* has been reported to control GA biosynthesis and activation genes [33, 39], we investigated whether the squash *myb62* mutation was also capable of altering the GA pathway. Treatments of WT and *myb62* plants with GA3 and the GA biosynthesis inhibitor paclobutrazol altered the elongation of the internodes in both genotypes (Fig. S3) and the percentage of female flowers in WT plants but did not change the androecious sex phenotype of the mutant (Supplementary Table S7). These findings indicated that *CpMYB62* regulated sex determination independently of GA. However, our experimental data showed that *CpMYB62* realized its action through the mediation of ethylene: (i) Ethylene production was greatly reduced in the apical shoots of *myb62* plants (Fig. 3B). (ii) Exogenous application of ethylene application was able to partially rescue the androecious phenotype of the *myb62* mutant, converting androecious to monoecious plants that produce some female flowers (Fig. 3C and D). The interruption of treatment results in a reversion to the androecious phenotype, once the ethylene drops again to its internal level, which means that mutant plants are able to perceive and respond to ethylene but have blocked the ethylene-mediated production of female flowers (Supplementary Fig. S4). In this situation, CpMYB62 should act upstream of ethylene biosynthesis, otherwise mutant plants would not respond to ethylene application. (iii) The androecious phenotype of the double mutant *myb62/etr2b* indicates that *myb62* is epistatic over the ethylene receptor gain-of-function mutation *etr2b*. Contrary to what was observed for *myb62* plants, where the mutation completely blocked the formation of female flowers, the *etrb2b* mutation confers partial insensibility to ethylene, leading to an andromonoecious phenotype [19, 20]. Since *myb62* is epistatic over *etr2b*, *it* should act upstream of *etr2b* and block ethylene biosynthesis in the double mutant leading to androecy instead of the andromonoecy phenotype of *etr2b*. Together, these results showed that *CpMYB62* positively controls ethylene production during the specification of female flowers.

Different mutations in ethylene biosynthesis and perception genes have been reported to convert monoecy into andromonoecy or androecy in squash, melon, and cucumber [12, 13, 18–20, 40]. Since ethylene production was reduced in the apical shoots of *myb62*, and ethylene rescued the mutant phenotype, it is likely that CpMYB62 regulates gene transcription in the ethylene biosynthesis pathway rather than in the ethylene perception or signaling pathway. The fact that *myb62* did not change plant ethylene sensitivity (Fig. 3A), nor did it change the expression of ethylene perception and signaling genes (Fig. 5C) also supports this conclusion. In fact, the comparative study of apical shoot transcriptomes at the stage of female flowering clearly showed that the *CpMYB62* gene upregulates *CpACO2* and *CpACS27A* (Fig. 5A), two key ethylene biosynthesis genes in the control of cucurbit sex determination. The ethylene produced by the enzyme CpACO2 promotes the development of carpel [13, 18] while that produced by the enzyme CpACS27 is responsible for stamen arrest during the specification of female flowers [14–17]. Moreover, the ethylene-regulated transcription factors CRC and HB40, which have recently been reported to promote the development of carpels and arrest the development of stamens in melon and cucumber [28, 29] were also upregulated by CpMYB62. As expected, *CpWIP1*, a master transcription factor gene that promotes male flower development in cucurbits [24–26], was downregulated by CpMYB62 in the female floral buds of the apical shoot. However, CpMYB62 was not found to regulate ethylene receptors (*CpETR1A*, *CpETR1B*, *CpETR2B*) and signaling genes (*CpEIN3A*, *CpEIN3B*, *CpEIL1A*, and *CpEIL1B*) (Fig. 5B), although the expression of the latter was also associated with the presence of female flowers primordia. Taken together, these findings demonstrate that *CpMYB62* not only regulates ethylene biosynthesis genes but also several master sex-determining transcription factors such as CpCRC, CpHB40, and CpWIP1. And it is likely that downregulation of genes in the ethylene biosynthesis pathway and genes coding for transcription factors that act downstream of ethylene is what causes the lack of female flowers in *myb62*. Interestingly, the expression of *CpMYB62* was completely lost in the apical shoots of the *myb62* mutant, similar to what occurs in the androecious ethylene insensitive mutant *etr1b* [23]. This suggests that there is a positive feedback regulation of *CpMYB62* that is also likely mediated by ethylene. This conclusion is also supported by the predicted ethylene response elements presented on the promoter of *CpMYB62* (Fig. 4D).

Other cucurbit sex determining genes were not found to be regulated by the transcription factor CpMYB62. The ethylene biosynthesis gene *CpACS11*, which, together with *CpACO2*, is responsible for promoting the development of carpels in female flowers, was not found to be differentially expressed in the apical shoots of WT and *myb62* plants upon female flowering. No significant difference was either observed in the expression of *CpbZIP48, CpTPLA, CpTPLB,* and *CpCUC2B* between the apical shoots of WT and *myb62* at the female flowering stage of development. The melon *CmbZIP48* and *CmTPL* contribute to maleness by interacting with *CmWIP1* [28, 41], but are expressed in both male and female primordia. *CpCUC2B* also enhances maleness in *C. pepo* by arresting carpel development independently of ethylene and *CpWIP1* and by promoting stamen development through the downregulation of *CpACS27A* [30]. Since many of these genes are also positively or negatively regulated by ethylene [23], the fact that they escape the regulation by *CpMYB62* could indicate that *CpMYB62*-mediated ethylene production occurs in a specific tissue or stage of development of the female flower, different from that which regulates the transcription of these genes.

All these findings have been integrated into a model that explains the gene interactions controlling sex determination in cucurbit species (Fig. 6). The novel transcription factor gene *CpMYB62* is the first reported gene that functions upstream of ethylene production in female floral buds at the earliest stages of development, downregulating so the expression of *CpWIP1* and the subsequent activation of *CpACS27* that lead to the specification of a female flower. In the absence of *CpMYB62*, ethylene is not activated, and the upregulation of *CpWIP1* leads to the arrest of carpel development, and the downregulation of *CpACS27* which results in the specification of a male flower.

## Material and methods

### Plant material origin and populations of analysis

123 M2 lines of the *C. pepo* EMS mutant collection [31] were grown to maturity in Almeria, Spain, during the spring–summer 2020 season under standard greenhouse conditions. The phenotypic screening led to the detection of a new mutant line (*myb62*) lacking female flowering transition. This line produced only male flowers.

The M2 androecious mutant plants were crossed with a scallop line (UPV-96), a different genetic background to generate F1 and F2 populations. In parallel, mutant plants were crossed with MU-CU-16, the background of the mutant collection, to generate the BC1 and BC2 generations as well as their selfing progenies BC1S1 and BC2S1. The F2 was used to perform Bulk Segregant Analysis Sequencing (BSA-seq), and the F2, BC1S1, and BC2S1segregating populations were then used to verify the causal mutation of the androecious phenotype by individual plant genotyping. All generations were phenotyped for sex expression, annotating the presence of male and female flowers in at least 60 nodes of the plant.

### Identification of *myb62* causal mutation by BSA-seq analysis coupled with WGS

To identify the mutation responsible for *myb62* androecious phenotype, two independent bulks of wild-type (WT) and *myb62* plants derived from the F2 segregating population were subjected to whole-genome sequencing (WGS) as was previously described in [30], using 25 plants to construct each bulk.

QTL-seq analysis was performed using the QTLseqr R package [42] with the following parameters: 7 < total read depth < 200, reference allele frequency (AF) <0.3, and genotype quality >25. The function “runQTLseqAnalysis” [43] was used to detect obtaining SNP-index and ΔSNP-index, using a 1 Mb sliding window, and the confidence intervals (95% and 99%) for the ΔSNP-indexes were determined using 10 000 simulations for each bulk.

SNPs present in the putative QTLs obtained were filtered according to the following parameters: alternative AF in the WT bulk <0.3, AF = 1 in the *myb62* bulk; read depth ≥ 7; genotype quality ≥30. Common SNPs derived from UPV-196 and between different EMS lines in the collection were discarded. Positions bearing canonical EMS mutations (G > A or C > T transitions) were selected as potential causal mutations and subjected to fine mapping analysis to detect the mutation responsible for the phenotype.

The segregation between *myb62* phenotype and the candidate SNPs was finally performed in 471 plants from segregating populations F2 and BC2S1. Genotyping was performed by Kompetitive allele-specific PCR (KASP) technology according to manufacturer’s instructions in the CFX96 Touch real-time PCR Detection System (Bio-Rad®).

### Bioinformatic analysis: Phylogeny and protein stability

Protein homologous to CpMYB62 were identified using the BLAST tool at NCBI (http://www.blast.ncbi.nlm.nih.gov) and synteny blocks were analyzed in CuGenDB v2 (http://cucurbitgenomics.org/v2/ [44],). The phylogenetic tree of identified MYB proteins in *C. pepo* and Arabidopsis (Supplementary Table S9) was constructed using Molecular Evolutionary Genetics Analysis (MEGA X) software [45] with Multiple Sequence Comparison by Log-Expectation (MUSCLE) alignment [46] and the Maximum Likelihood method based on the Poisson correction model [47], with 1000 bootstrap replicates to verify the reliability of the obtained tree.

Tertiary structure of the CpMYB62 WT and mutant protein was predicted with SWISS-MODEL resource [48].

### Transcriptomic and gene expression analysis

The expression of *CpMYB62* was obtained by (i) RNA sequencing (RNA-seq) of different plant organ samples in the genetic background MU-CU-16 previously obtained by our research group, and (ii) by RNA-seq of apical shoot samples from WT and *myb62* plants, and (iii) by quantitative RT-PCR of WT and *myb62* apical shoot samples.

Samples derived from WT and *myb62* plants grown in a culture chamber with a photoperiod of 16/8 h day/night and 25°C. Apical shoots with less than 1 mm (containing small leaves and floral meristems <0.5 mm) were collected at two different stages of plant development: M0: plants having 2 leaves (before FFT), M1: plants with 12–14 leaves (after FFT in WT).

RNA was extracted by the E.Z.N.A.® Plant RNA Kit (Omega Bio-tek, USA, R6827-01) following the manufacturer’s protocol. RNA-seq samples were sequenced by BGI Genomics using the DNBseq platform, generating 150 pb pair-end reads and a total of 6 Gb of raw data per sample. Analysis of raw data was performed as described in [23]. After analysis, BALLGOWN package in R [49] was used to obtain fragments per kilobase of transcript per million mapped reads (FPKM) values. Generated FPKMs were used to create heatmaps with TBtools [50].

For qRT-PCR, RNA was converted into cDNA with the cDNA RevertAid™ kit (Thermo Fisher Scientific®), and reactions were performed in 10 μL total volume with 1× Top Green qPCR Super Mix (Bio-Rad ®) in the CFX-96 Touch Real-Time PCR Detection System thermocycler (Bio-Rad®). The relative gene expression was calculated using the 2^–ΔΔCT^ method [51] with *CpEF1α* and *CpACT* genes as internal reference. Primers for qRT-PCR are shown in Supplementary Table S10.

### Exogenous hormone application and ethylene sensitivity assay

The androecious phenotype of the *myb62* mutant was attempted to be rescued by external application of paclobutrazol (inhibitor of GAs) or ethylene. The apical shoots of at least 10 WT and 10 *myb62* plants with 2–3 leaves were treated with 100 μl of 3 mM gibberellic acid (GA3), 500 ppm paclobutrazol, and water as control, all supplemented with 0.1% Tween-20. The duration of the treatment was 4 weeks, with one application per week. For the ethylene treatment, 30 WT and 30 *myb62* plants with four true leaves were introduced into a growth chamber containing 0 (Ct), and 100 ppm of ethylene for 2 days, and then transferred to a greenhouse under standard conditions for 3 weeks. The treatment was repeated three times with one application per week. WT and *myb62* plants were evaluated for sex expression traits after three weeks of growing.

To evaluate the sensitivity of plants to ethylene, the triple response test of etiolated seedlings and the flower abscission assay were performed as described in [31].

### Ethylene production measurements

Ethylene production was assessed in apical shoots of 2 cm from WT and *myb62* adult plants of 40 nodes growth under standard greenhouse conditions. Samples were collected and incubated at room temperature for 10 h in sealed glass containers of 50 ml. Ethylene production was analyzed in 1 ml of gas from the container in a Varian® 3900 gas chromatograph (Varian, USA), with flame ionization detector (FID). Calibration of the instrument was performed with standard ethylene gas. Three biological replicates and three measurements were taken per sample. Ethylene production was expressed as nl * g^−1^ FW * 10 h^−1^.

### Statistical analyses

An analysis of variance (ANOVA) was used to assess the statistical significance of the data in the statistical software Statgraphic Centurion XVIII. Differences between samples were separated by the least significant difference at a significance level of *P* ≤ 0.05.

## Supplementary Material

Web_Material_uhae115

## Data Availability

All relevant data can be found within the manuscript and its supporting materials. All the raw reads generated in this study have been deposited in the public database of the National Center of Biotechnology under BioProject Number PRJNA1018819 and PRJNA1042934.
