# Supplementary material for: The transcription factor CpMYB62 controls the genetic network that leads to the determination of female flowers in Cucurbita pepo
Source: Hortic Res. 2024 Apr 22;11(6):uhae115. doi: 10.1093/hr/uhae115 (PMC11197297; doi:10.1093/hr/uhae115)
Supplement: Web_Material_uhae115 [file web_material_uhae115.zip › Supplementary figures MYB review.pptx]

## Slide 1
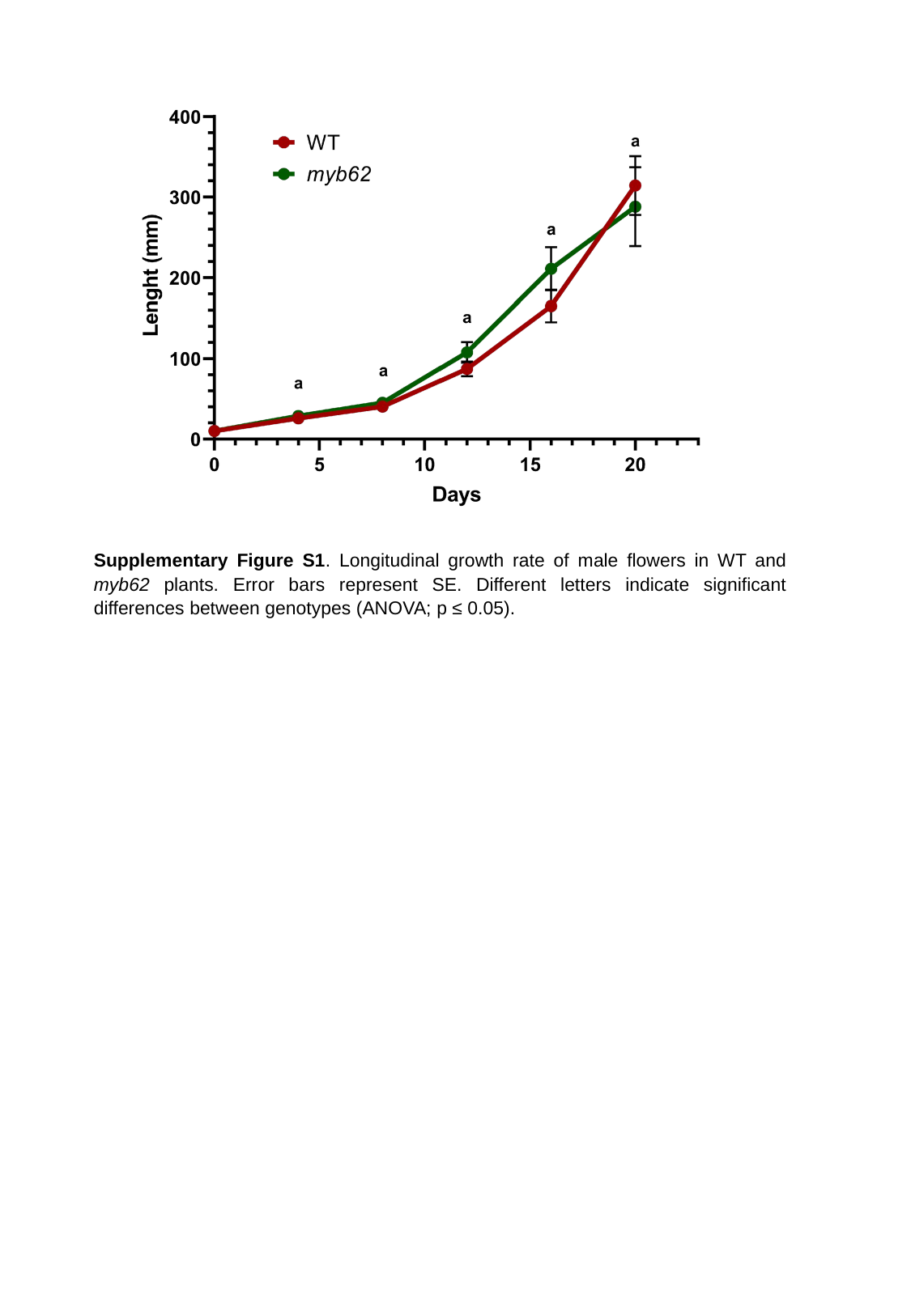

Supplementary Figure S1. Longitudinal growth rate of male flowers in WT and myb62 plants. Error bars represent SE. Different letters indicate significant differences between genotypes (ANOVA; p ≤ 0.05).

## Slide 2
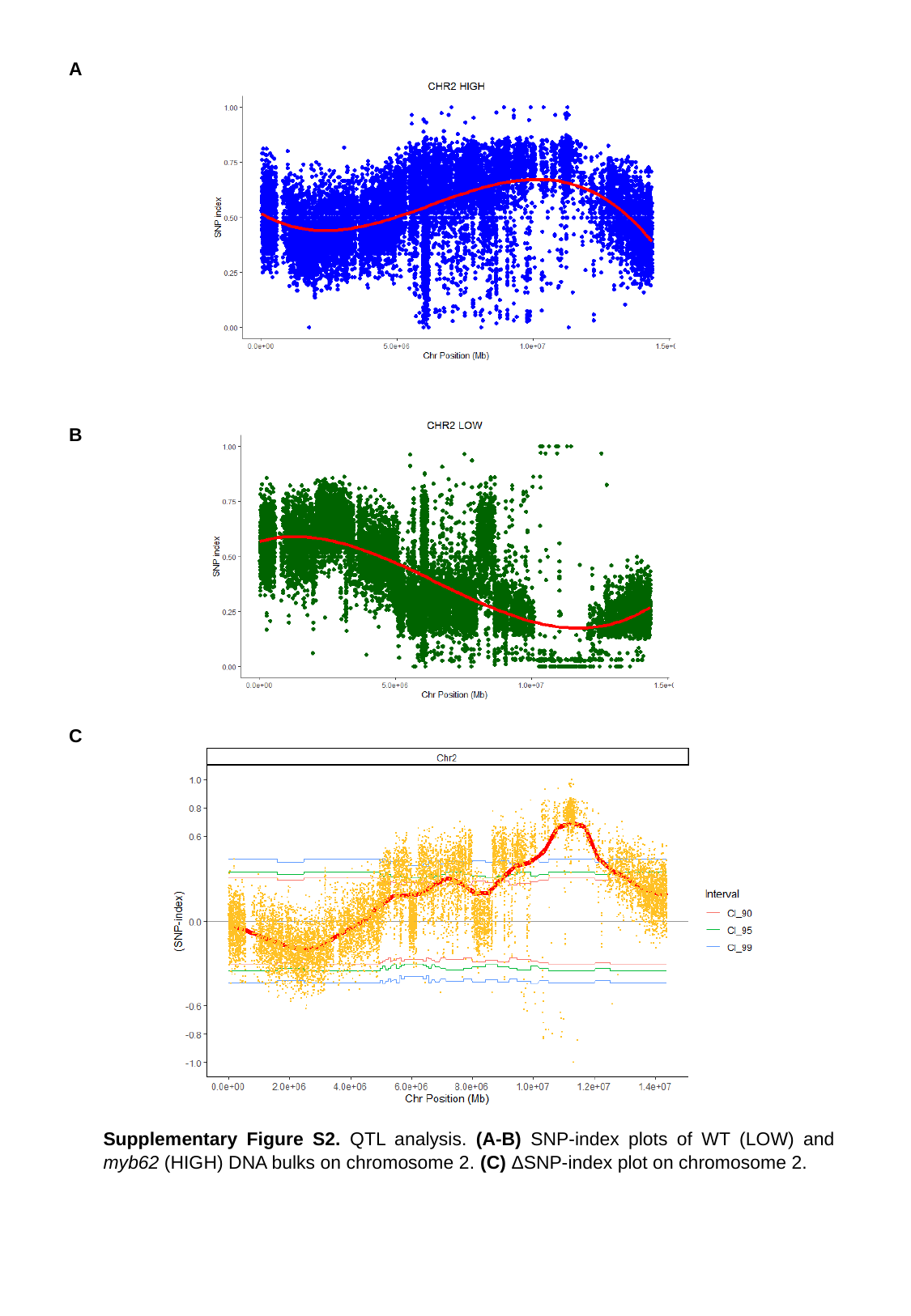

A
B
C
Supplementary Figure S2. QTL analysis. (A-B) SNP-index plots of WT (LOW) and myb62 (HIGH) DNA bulks on chromosome 2. (C) ΔSNP-index plot on chromosome 2.

## Slide 3
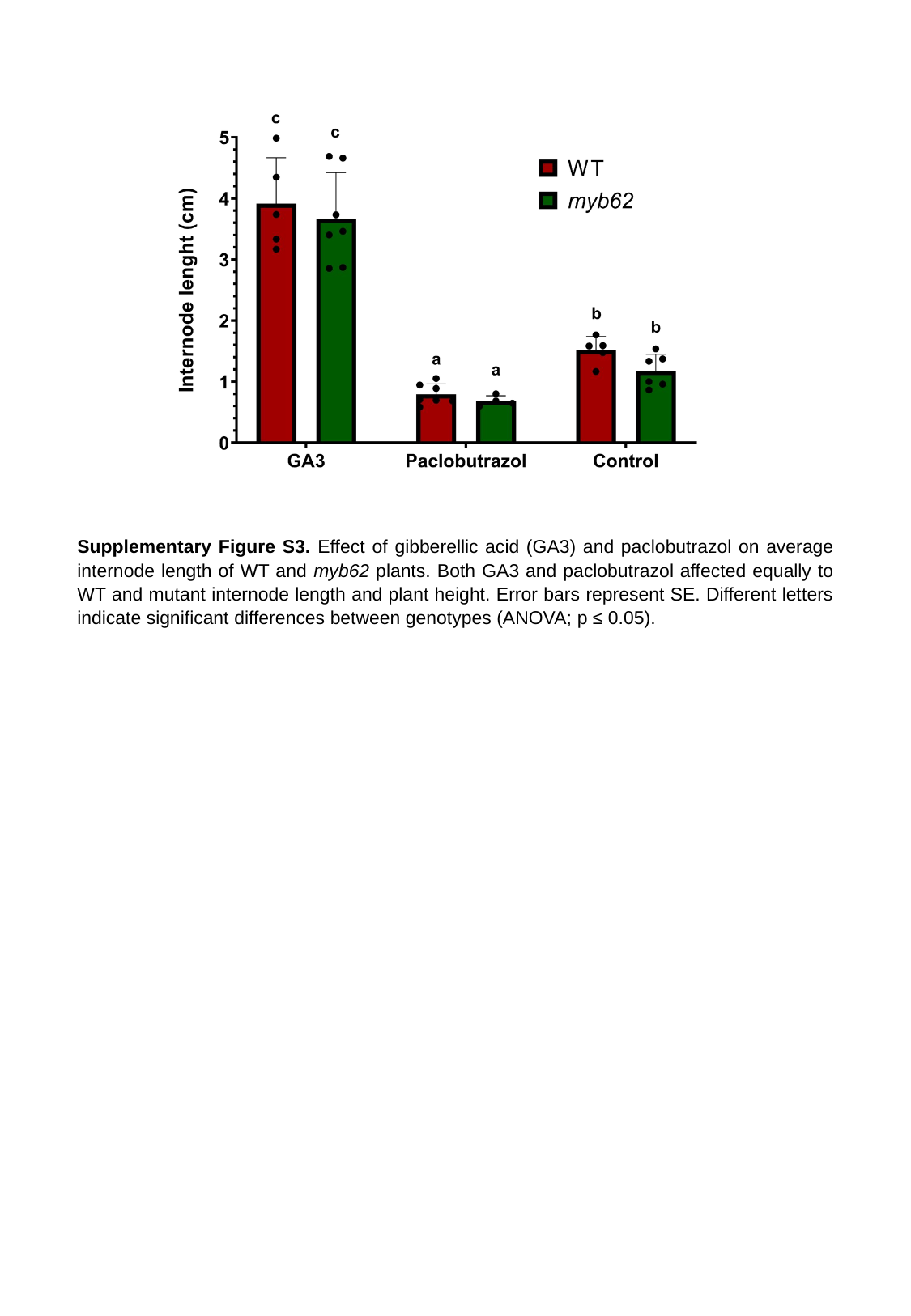

Supplementary Figure S3. Effect of gibberellic acid (GA3) and paclobutrazol on average internode length of WT and myb62 plants. Both GA3 and paclobutrazol affected equally to WT and mutant internode length and plant height. Error bars represent SE. Different letters indicate significant differences between genotypes (ANOVA; p ≤ 0.05).

## Slide 4
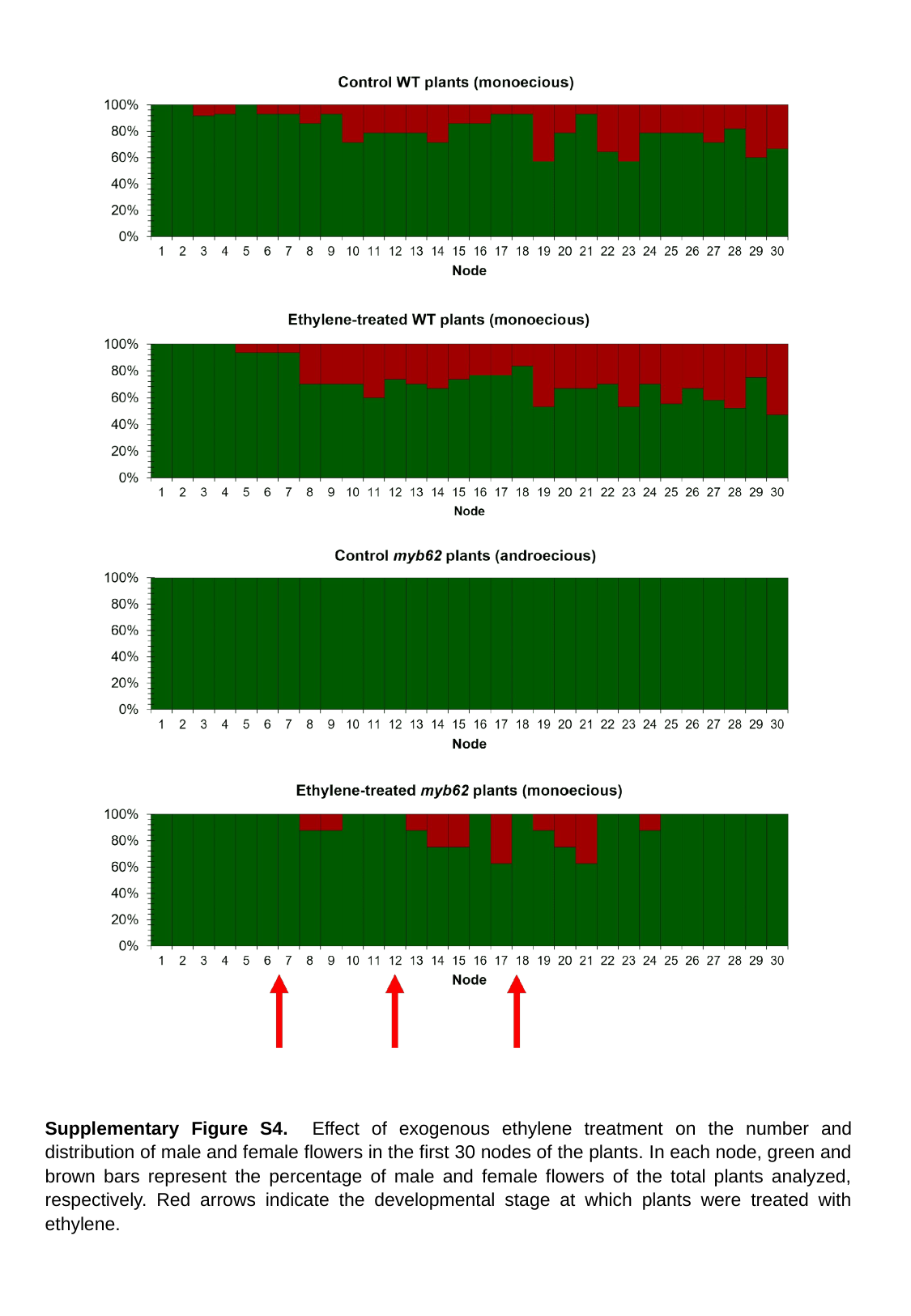

Supplementary Figure S4. Effect of exogenous ethylene treatment on the number and distribution of male and female flowers in the first 30 nodes of the plants. In each node, green and brown bars represent the percentage of male and female flowers of the total plants analyzed, respectively. Red arrows indicate the developmental stage at which plants were treated with ethylene.

## Slide 5
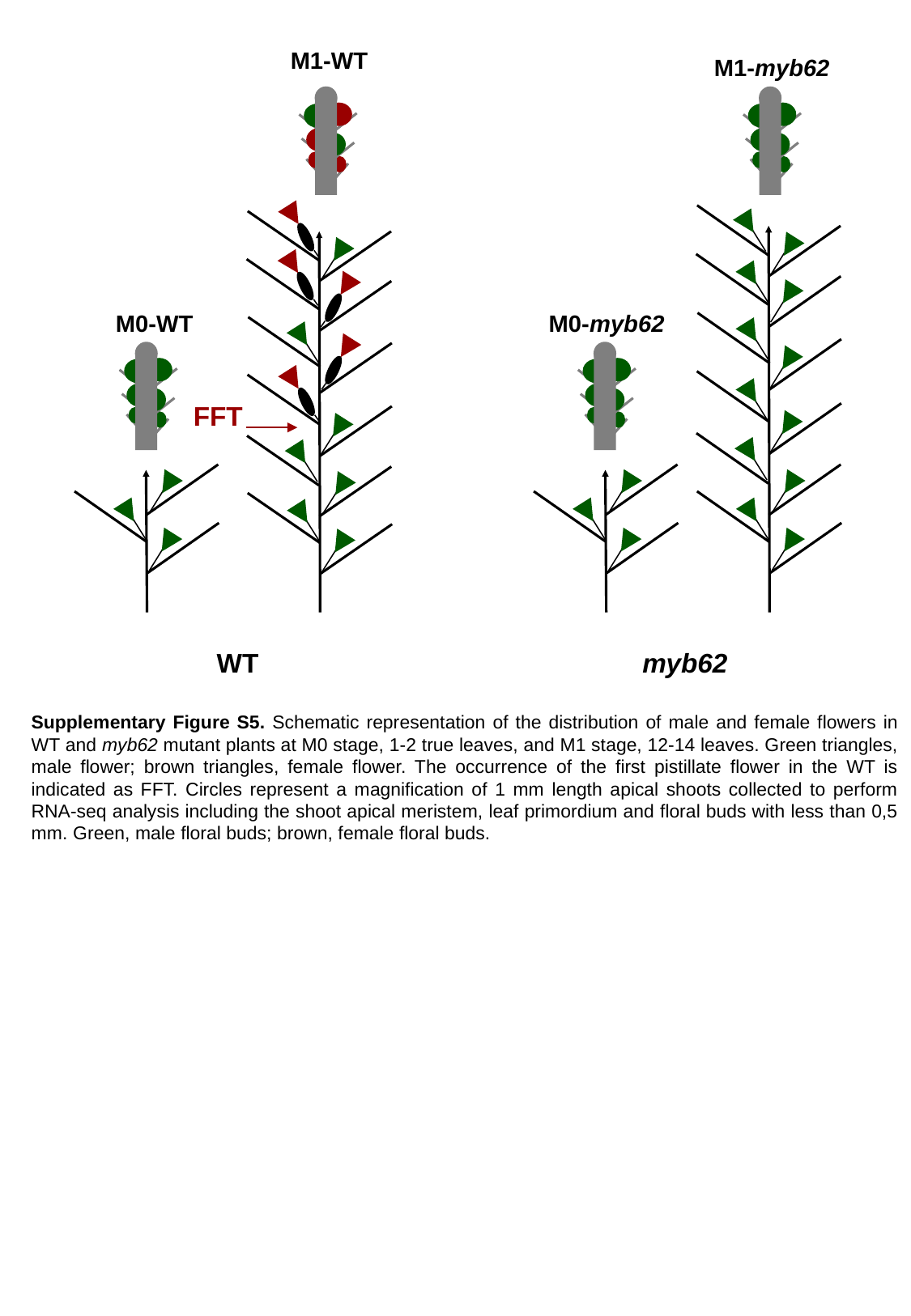

M1-WT
M1-myb62
M0-myb62
M0-WT
FFT
WT
myb62
Supplementary Figure S5. Schematic representation of the distribution of male and female flowers in WT and myb62 mutant plants at M0 stage, 1-2 true leaves, and M1 stage, 12-14 leaves. Green triangles, male flower; brown triangles, female flower. The occurrence of the first pistillate flower in the WT is indicated as FFT. Circles represent a magnification of 1 mm length apical shoots collected to perform RNA-seq analysis including the shoot apical meristem, leaf primordium and floral buds with less than 0,5 mm. Green, male floral buds; brown, female floral buds.

## Slide 6
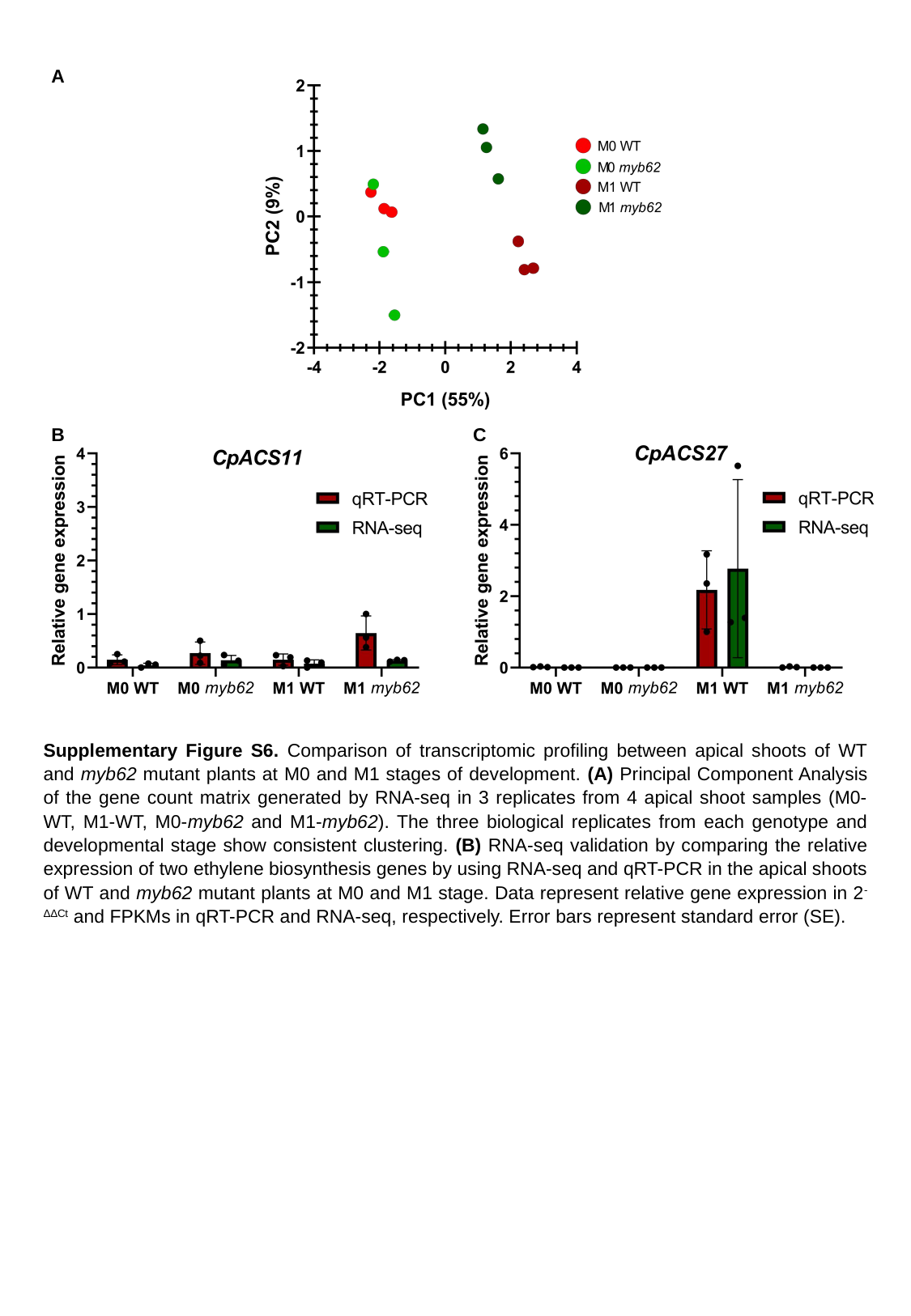

A
B
C
Supplementary Figure S6. Comparison of transcriptomic profiling between apical shoots of WT and myb62 mutant plants at M0 and M1 stages of development. (A) Principal Component Analysis of the gene count matrix generated by RNA-seq in 3 replicates from 4 apical shoot samples (M0-WT, M1-WT, M0-myb62 and M1-myb62). The three biological replicates from each genotype and developmental stage show consistent clustering. (B) RNA-seq validation by comparing the relative expression of two ethylene biosynthesis genes by using RNA-seq and qRT-PCR in the apical shoots of WT and myb62 mutant plants at M0 and M1 stage. Data represent relative gene expression in 2-ΔΔCt and FPKMs in qRT-PCR and RNA-seq, respectively. Error bars represent standard error (SE).
